# Hospital admission is associated with disability and late musculoskeletal pain in individuals with long COVID

**DOI:** 10.3389/fresc.2023.1186499

**Published:** 2023-10-27

**Authors:** Ricardo Bezerra Duarte Neto, Luis Felipe Fonseca Reis, Arthur de Sá Ferreira, Dângelo José de Andrade Alexandre, Renato Santos de Almeida

**Affiliations:** ^1^ Post Graduation Program in Rehabilitation Sciences, Augusto Motta University Center (UNISUAM), Rio de Janeiro, Brazil; ^2^ Physiotherapy Department. Rio de Janeiro Military Police Rehabilitation Center (CFRPM-RJ), Rio de Janeiro, Brazil; ^3^ Physiotherapy Department. Rio de Janeiro Ortophaedic Trauma Institute (INTO), Rio de Janeiro, Brazil; ^4^ Physiotherapy Department. Serra dos Órgãos University Center (UNIFESO), Teresópolis, Brazil; ^5^ Physiotherapy Department, Rio de Janeiro Federal Institute (IFRJ), Rio de Janeiro, Brazil

**Keywords:** long-COVID, musculoskeletal pain, disability, hospitalization, physical therapy

## Abstract

**Background:**

The acute clinical repercussions of SARS-CoV-2 infection have been widely studied. However, the possible late repercussions of long COVID have not yet been well defined in the literature.

**Objectives:**

To identify the presence of pain and musculoskeletal disability in patients with Long COVID and also to identify predictive factors for pain intensity in this population.

**Methods:**

In this cross-sectional and retrospective observational study individuals with Long COVID symptoms were included. It was collected musculoskeletal disability measures, data from patient-related outcome measures and variables from a COVID-19 outpatient service database. Associations and sub-group analyses were performed considering the variables pain, disability and hospitalization. Linear regression was performed to identify predictive factors for pain intensity in Long COVID patients.

**Results:**

We evaluated 195 patients and most of them (57%) presented musculoskeletal pain in one area of the body. Pain sub-group presented worse disability indices and worse clinical course during hospitalization. Hospitalized patients presented worse disability indices comparing to non-hospitalized. Significant correlations were found between pain and days of non-invasive oxygen support (*r* = 0.21; *p* = 0.003); days in intensive care unit (*r* = 0.22; *p* = 0.002) and days in invasive mechanical ventilation (*r* = 0.35; *p* = 0.001). Hospitalized individuals showed a higher chance of presenting late musculoskeletal pain (OR = 1.42: 95%CI 1.09–2.04). Days in intensive care unit (*β* = 0,234: *P* = 0,001) and days in invasive mechanical ventilation (*β* = 0.764: *P* = 0.001) were predictors of pain intensity [F(2,192) = 18.559; *R*^2^ = 0.231; *p* = 0.001].

**Conclusion:**

Individuals with Long COVID presented musculoskeletal pain and disability. Hospitalized patients showed a greater chance of having musculoskeletal pain. Days in intensive care unit and days in invasive mechanical ventilation were predictors of late musculoskeletal pain intensity.

## Introduction

The acute clinical repercussions of SARS-CoV-2 infection have been widely studied since the beginning of the pandemic scenario (1–3). However, the possible repercussions of COVID long-term effects are not yet well defined in the literature (1–5). Considering this phenomenon the World Health Organization (WHO) defined the term Long COVID as a health condition that occurs in individuals with a history of probable or confirmed SARS-CoV-2 infection, usually three months after this diagnosis, with symptoms not explained by other possible diagnoses that persist for at least two months (6–8). The prevalence of Long COVID is still controversial in the and also the main symptoms that could be associated with this health condition. Data from systematic reviews indicate that 25% of individuals who contracted the COVID-19 virus continue to have symptoms after at least one month of diagnosis (4, 9–12). The incidence of Long COVID after hospital discharge varies from 2.3% to 80% (9, 13–15). The meta-analysis conducted by Lopez-Leon identified more than 50 COVID long-term symptoms and demonstrated that 80% of infected patients developed at least one of these symptoms (16). Among the most common Long COVID symptoms, peripheral fatigue, dyspnea on moderate activities, joint pain, and cognitive problems can be high lightened (4, 6, 17–23).

Musculoskeletal manifestations in COVID-19 have being studied by different researches groups (13, 16–19). Clinicians should consider that musculoskeletal manifestations probably have been often underreported. Due the fact that patients who most frequently present musculoskeletal pain complaint during hospitalization are more severely ill and/or sedated other clinical outcomes are usually evaluated (16). However, the incidence of thrombotic events possibly associated with the clinical profile of COVID-19 patients may also contribute with poor vascularization of peripheral vessels in the muscles (4) and might generate biochemical repercussions in the musculoskeletal system. The pathogenetic mechanisms can also involve increased production of proinflammatory cytokines, immune cell hyperactivation, direct viral entry of neurological and musculoskeletal system cells, and psychological stress (18–25). The cognitive and behavioral aspects may be also associated with the occurrence of musculoskeletal pain in these patients (18–22). Considering specifically joint pain, some findings show a prevalence that may vary between 2.5% and 23% of patients (21–25). Myalgias and generalized weakness have also been reported in studies with a prevalence between 25% and 50% of symptomatic COVID-19 patients (23). Fatigue is another symptom widely mentioned in the literature as well as cognitive problems, psychological stress, and shortness of breath (26–32).

The investigation of the possible variables associated with musculoskeletal pain in Long COVID patients is still incipient and the causal relationships for musculoskeletal pain in these patients are not yet well established in the literature. The discussion about variables that may help in the construction of explicative models for pain occurrence in Long COVID patients should be stimulated in different populations. Thus, the aim of this study were to identify the presence of pain and Musculoskeletal disability in patients with Long COVID and also to identify predictive factors for pain intensity in this population.

## Methods

### Study design

This cross-sectional and retrospective observational study included musculoskeletal disability measures, patient-related outcome measures and database analysis. The study has been carried out following the Helsinki Declaration and obtained approval from the Research Ethics Committee at Centro Universitário Augusto Motta (UNISUAM). It was approved under the number CAAE 48074921.7.0000.5235. The informed consent was presented to all patients, and it must be signed before data collection. This research report followed the Strengthening the Reporting of Observational Studies in Epidemiology (STROBE) statement.

### Settings and participants

Participants were recruited through face-to-face assessments. Patients who attended the Post-COVID-19 Outpatient Service of the Rio de Janeiro Military Police Physiatrics and Rehabilitation Center (CFRPM-RJ) between July 2020 and March 2022 were invited to participate. Patients were referred after a medical consultation at the Central Hospital of the Rio de Janeiro Military Police (HCPM-RJ). The CFRPM-RJ is a Military Police health unit that has an outpatient service and monitors all patients who had at least one medical consultation due to COVID-19 symptoms with or without a hospitalization history.

The following inclusion criteria were considered: (1) individuals over 18 years old; (2) more than 90 days of positive COVID RT-PCR test, considering the moment of enrollment; and (3) individuals who had any musculoskeletal pain complaint that did not exist before the diagnosis of COVID-19. Exclusion criteria were: (1) patients with any surgical intervention for at least 3 months before diagnosis; (2) individuals undergoing current cancer treatment; (3) individuals with a history of walking limitations before diagnosis; and (4) individuals with cognitive or functional alterations that made it impossible to perform the tests.

The sample size calculation considered a model for regression analysis with 3 possible predictors of pain intensity (main outcome) in the GPower 3.1 Software. Because there is no consensus in the literature regarding the possible associated or predictive variables of late pain in this patient profile, the sample size calculation considered an effect size of 0.10, with a statistical power of 80% and an error of 5%. The calculation showed the necessity to include 176 participants in the research. The sample size calculation for the other analyses of possible associations between the variables required entering a smaller number of participants than the number of patients that were included for regression analysis.

### Measurement instruments and procedures

The outpatient evaluation collected sociodemographic and clinical data from the participants (age, body mass index, comorbidities, and COVID-19 diagnosis date). The patients were asked at the moment of the assessment if they had been hospitalized and the patient’s medical record was consulted in the hospitalization sector. The following data related to hospitalization were collected: days of hospitalization, days in non-invasive oxygen support, days in intensive care unit (ICU) and days in invasive mechanical ventilation (MV).

The following measurement instruments were applied in order to identify pain intensity and musculoskeletal disability: the Numerical Pain Rating Scale (NPRS); Fatigue Pictogram; Berg Balance Scale; the Post COVID Functional Scale (PCFS) and the 10-meter Walk Test. All evaluations were performed by a single evaluator, a physiotherapist, with more than 15 years of experience in the area of musculoskeletal rehabilitation, under the supervision of a senior researcher with experience in clinical research.

Pain assessment was performed using the Numerical Pain Rating Scale (NPRS). This is a simple 11-point scale with data that is easy to document and interpret and is very useful in pain assessment, graded from 0 (zero)—no pain, to 10 (ten)—worst pain (33, 26).

Fatigue assessment was performed using the Fatigue Pictogram (34). Pictograms are tools that use illustrations and, therefore, are easy to understand and have excellent clinical applicability (35). The selected instrument is an ordinal scale composed of two sets of five figures that assess the intensity of fatigue and the degree of limitation that it entails in the individual’s daily activities (34, 35). The first series of figures answers the following question “How tired have you felt in the last week?” and the second series answers the question “How much does the feeling of tiredness prevent you from doing what you want to do?”. The instrument has formally gone through a cross-cultural adaptation process for Brazil (35). In our work, we assigned numbers to each image to help with the quantitative analysis of the data.

Regarding the assessment of balance and risk of falling, we used the Berg Balance Scale (36). The instrument can be self-administered, and assesses the individual’s functional balance through 14 items related to activities of daily living (36, 37). Items are based on the duration of time the individual can maintain a position, the arm’s reach distance in front of the body, and the time to complete each task. Each item corresponds to a scale graduated from 0 (zero) to 4 (four) points, with a maximum score of 56 points: scores between 53 and 46 indicate a risk of falling from mild to moderate; scores below 46 points indicate a high risk of falling (37, 38).

The Post-COVID-19 Functional Status Scale was also applied to investigate disability (39). This instrument was proposed as a way of measuring the consequences of COVID-19 and its impact on the functional status reported by the patient and can be applied at the time of hospital discharge and later to monitor post-discharge conditions (38). The scale is graded in six levels, from “no functional limitations” (zero grade) to “severe functional limitations” (grade 4) and “death” (grade 5) (39, 40).

The 10-meter Walk Test was also applied. It consists in timing a free ten meters of walking in the usual speed (41). Starting from the orthostatic position the initial two meters and the final two meters are added just to consider the moments of acceleration and deceleration respectively (41, 42).

### Data analysis

The Shapiro-Wilk tests suggested a normal distribution of variables. The clinical profile and characteristics of the sample were described using means and standard deviation or occurrence frequency (percentage).

Two subgroup analyses were performed: one comparing disability and pain intensity means from hospitalized and non-hospitalized individuals; and another one comparing disability and data hospitalization means from patients with and without pain. The Student´s *t*-test for two independent samples was applied for these analyses.

Possible associations between disability variables, hospitalization data and pain intensity were evaluated. Pearson’s coefficient correlation was applied to identify possible correlations between continuous variables. The odds ratio was used to quantify associations for categorical variables.

To identify predictive factors for pain a linear regression analysis was performed. The model considered pain intensity as a dependent variable and days of hospitalization, days in intensive care unit and days in invasive mechanical ventilation support as independent variables. The model was built using the backward regression method, starting from a model with all variables and eliminating non-significant variables. Data were analyzed using the JASP Version 16.3 assuming a significance level of 5%.

## Results

A total of 195 participants were included in the study. The population had a mean age of 54.74 years (SD 11.12), 137 (70%) were males, 58 (29%) were females and the BMI mean was 35.31 kg/m^2^ (DP 4.8). We observed that 121 (62%) participants presented systemic arterial hypertension, 87 (45%) were diabetic, 35 (18%) were smokers, and most of the sample (*n* = 165) was hospitalized due to COVID-19 (85%). Most patients, 112 (57%), reported musculoskeletal pain in one region of the body at the moment of the evaluation and 83 patients (43%) presented pain complain in 2 or more body areas. The average of data collect presented 120 days (SD 2) after COVID-19 diagnosis. Table 1 presents the sample characteristics and mean values of disability measures collected.

**Table 1:** Sample characteristics and disability measures collected (*N* = 195).

Variables	Mean ± SD; (%)
Age (years)	54.7 ± 11.1
BMI(Kg/cm^2^)	35.3 ± 4.8
Sex (males; %)	70%
Comorbidities	
Systemic arterial hypertension	62%
Diabetes	45%
Pain complain	57%
Fatigue Pictogram (Total score)	3.0 ± 1.1
Gait Speed (m/s)	1.6 ± 0.7
PCFS (Total score)	2.6 ± 0.9
Berg Scale (total score)	53.9 ± 2.3
NPS (Total score)	2.2 ± 2.3

BMI, body mass index; NPS, numerical pain scale; PCFS, post covid functional status scale.

Comparing the sub-groups of hospitalized patients (*n* = 165) and non-hospitalized patients (*n* = 30) significant differences were also found in the means of pain intensity and disability variables collected. The results showed that the hospitalized patients presented worse disability indices and more pain. The means of these both groups are shown in Table 2. Because we found no statistical evidence of difference between sub-groups for age, sex and body mass index the groups were considered as homogeneous.

**Table 2:** Mean values of pain and disability measures collected for hospitalized and non-hospitalized patients.

Variables	Hospitalized (*n* = 165) (mean ± SD)	Non-hospitalized (*n* = 30) (mean ± SD)	*P*-value
Berg Scale	50.5 ± 3.7	53.4 ± 2.6	0.04
PCFS	2.5 ± 0.9	3.0 ± 0.8	<0.01
Fatigue	3.1 ± 1.1	2.6 ± 0.8	0.04
Gait Speed (m/s)	1.5 ± 0.6	2,0 ± 0.9	<0.01
NPS	2.3 ± 2.4	1.8 ± 2.4	0.03

PCFS, post covid functional status scale; NPS, numerical pain scale.

Comparing the sub-groups patients with pain (*n* = 112) versus patients without pain (*n* = 83) significant differences were found in disability variables means and also in the hospitalization data collected. The results showed that the group with pain presented worse disability indices and worse clinical course during hospitalization. The means of both groups are show in Table 3. Because we found no statistical evidence of difference between sub-groups for age, sex and body mass index the groups were considered as homogeneous.

**Table 3:** Mean values of disability measures and hospitalization data for groups with and without pain.

Variables	Group With Pain (*n* = 112)	Group Without Pain (*n* = 83)	*P*-value
Days in Intensive Care Unit	18.7 (±13.1)	11.2 (±12.1)	<0.01
Mechanical Ventilation days	16.4 (±12.2)	7.1 (±11.8)	<0.01
Days with oxygen support	22.0 (±13.6)	14.4 (±12.2)	<0.01
Berg Scale (total score)	51.7 (±3.9)	54.3 (±2.8)	<0.01
PCFS (total score)	3.0 (±0.8)	1.8 (±0.8)	<0.01
Fatigue (total score)	3.4 (±1.1)	1.7 (±1.4)	<0.01
Gait Speed (m/s)	1.5 (±0.7)	2.0 (±0.8)	<0.01

PCFS, post covid functional status scale.

The correlation analysis showed a positive correlation between mechanical ventilation duration and pain intensity (*r* = 0.346; *p* = 0.001). Thus, individuals with a longer duration of mechanical ventilation had greater pain intensity. A negative correlation was also observed between the value obtained on the Berg Scale and pain (*r* = −0.319; *p* = 0.001). Thus, a lower index on the Berg Balance Scale was associated to pain intensity. A correlation was also identified between the value on the scale and pain intensity (*r* = 0.381; *p* = 0.001). The higher the value on the PCFS score (indicating less functionality), the greater the pain intensity. The pain intensity also showed a correlation with the fatigue pictogram (*r* = 0.501; *p* = 0.00). Correlation analyses are described in Table 4.

**Table 4:** Correlations between disability measures, hospitalization data and pain intensity (*N* = 112).

Variables	Numerical Pain Scale (Pearson Coefficient- r)	*P*-value
Days in Intensive Care Unit	0.219	<0.01
Mechanical ventilation days	0.346	<0.01
Days with oxygen support	0.214	<0.01
Fatigue (total score)	0.501	<0.01
PCFS (total score)	0.381	<0.01
Gait Speed	−0.188	<0.01
Berg Scale (total Score)	−0.319	<0.01

PCFS, Post COVID Functional Scale.

The association analysis of pain and hospitalization reveled a significant relationship. It was found that hospitalized individuals have more chance to present late musculoskeletal pain when compared to non-hospitalized individuals (OR = 1.42; 95%CI 1.09–2.04).

The regression analysis used first an exploratory model, including pain intensity as a dependent variable and days of hospitalization, days in intensive care unit, and days in invasive mechanical ventilation support as independent variables. However, this model did not show statistical significance [F(3,192) = 11.357; *R*^2^ = 0.342; *P* = 0.13]. A second model considering the variables days in the intensive care unit and days in invasive mechanical ventilation support found statistical significance [F(2,192) = 18.559; *R*^2^ = 0.231; *P* = 0.001]. The independent variables showed low level of correlation but the model didn’t present multicollinearity. It was identified that the variables “days in intensive care unit” (*β* = 0.234; *P* = 0.001) and “days in invasive mechanical ventilation support” (*β* = 0.764; *P* = 0.001) were predictors of pain intensity.

## Discussion

The findings indicate that musculoskeletal pain had a high prevalence in the observed population with Long-COVID and participants with pain presented worse disability indices and worse disease clinical course during hospitalization. Hospitalized patients also presented worse disability indices. The odds ratio analysis in our sample showed that hospitalized patients had more chance to present late musculoskeletal pain when compared to non-hospitalized ones. Days in intensive care unit and days in invasive mechanical ventilation support were predictive variables for pain intensity.

The findings about the prevalence of late musculoskeletal pain in Long-COVID patients corroborate with studies in different populations from different parts of the world. A systematic review conducted by Akbarialiabad et al. reported a prevalence of 51% for late musculoskeletal pain in patients with more than 6 months after COVID-19 diagnosis (26). Soares et al. observed the occurrence of pain in COVID-19 patients after 16 weeks of hospital discharge and found that approximately 65% of individuals reported musculoskeletal pain (42).The age range and period after hospital discharge in which data were collected in the present study are similar to those in other studies (9, 10, 39, 40). This aspect favors the discussion about the main findings that have been reported in Long-COVID patients (14, 41–50). Our findings not only presented prevalence of pain in this specific population but also described some variables that are associated with pain intensity and possible predictors for late pain in the population included in the study.

Our findings high lightened the importance of continuous assessment of COVID-19 patients even after hospital discharge. The impact of Long-COVID on aspects such as gait, balance, and daily life activities should also be considered by health professionals. Similar findings were reported by Giardini et al. that found worse performance on static and dynamic balance measures in post-COVID-19 patients compared to healthy individuals (51). Pant et al. (52) reported that 46% of the population included in their study presented disability and quality of life complaints.

The finding regarding the predictive variables of musculoskeletal pain intensity is another important message for clinicians (9, 36). It reinforces the importance of preventive actions to minimize the complexity and the days of hospitalization (37). Nonetheless, measuring the real effect of COVID-19 on patients is quite difficult due to the lack of studies that investigate late musculoskeletal repercussions after hospitalization for “non-COVID causes”. However, Kosilek et al. (49) carried out an observational study in a hospital unit in Germany and demonstrated that 61% of patients with Post-Intensive Care Syndrome had chronic pain even 6 months after discharge. The prevalence of pain in patients with Post-Intensive Care Syndrome can reach 73% (50). COVID-19 patients seem to be susceptible to developing this syndrome (44) and this phenomenon makes it even more difficult to understand the consequences strictly related to COVID-19.

Our study has some limitations. We should face that the lack of a control group (non-COVID) does not allow us to isolate the real impact of COVID-19 on the participants. Data collection occurred in only one healthcare center and data analysis comparing different sample size sub-groups could have decreased the statistical power. However, the findings of some pain predictive variables in this study can collaborate with discussions about musculoskeletal complaints in Long-COVID and intensive care syndrome patients.

## Conclusion

Musculoskeletal pain had a high prevalence in the observed population with Long-COVID. Patients with pain presented worse disability indices and worse clinical course during hospitalization. Hospitalized patients showed more chance to present late musculoskeletal pain comparing to non-hospitalized ones. Days in intensive care unit and days in invasive mechanical ventilation support were predictive variables for pain intensity.

## Data Availability

The original contributions presented in the study are included in the article/Supplementary Material, further inquiries can be directed to the corresponding author.

## References

[1] Barker-Davies RM O’Sullivan O Senaratne KPP Baker P Cranley M Dharm-Datta S The Stanford Hall consensus statement for post-COVID-19 rehabilitation. Br J Sports Med. (2020) 54(16):949–59. 10.1136/bjsports-2020-102596 32475821 PMC7418628

[2] Crook H Raza S Nowell J Young M Edison P. Long COVID—mechanisms, risk factors, and management. Br Med J. (2021) 26:n1648. 10.1136/bmj.n1648 34312178

[3] Ochani R Asad A Yasmin F Shaikh S Khalid H Batra S COVID-19 pandemic: from origins to outcomes. A comprehensive review of viral pathogenesis, clinical manifestations, diagnostic evaluation, and management. Infez Med. (2021) 29(1):20–36. 33664170

[4] Ma Y Deng J Liu Q Du M Liu M Liu J. Long-term consequences of COVID-19 at 6 months and above: a systematic review and meta-analysis. Int J Environ Res Public Health. (2022) 19(11):6865. 10.3390/ijerph19116865 35682448 PMC9180091

[5] Premraj L Kannapadi NV Briggs J Seal SM Battaglini D Fanning J Mid and long-term neurological and neuropsychiatric manifestations of post-COVID-19 syndrome: a meta-analysis. J Neurol Sci. (2022) 434:120162. 10.1016/j.jns.2022.120162 35121209 PMC8798975

[6] Soriano JB Murthy S Marshall JC Relan P Diaz JV. WHO clinical case definition working group on post-COVID-19 condition. A clinical case definition of post-COVID-19 condition by a delphi consensus. Lancet Infect Dis. (2022) 22(4):e102–7. 10.1016/S1473-3099(21)00703-9 34951953 PMC8691845

[7] Gao P Liu J Liu M. Effect of COVID-19 vaccines on reducing the risk of long COVID in the real world: a systematic review and meta-analysis. Int J Environ Res Public Health. (2022) 19(19):12422. 10.3390/ijerph191912422 36231717 PMC9566528

[8] Chen C Haupert SR Zimmermann L Shi X Fritsche LG Mukherjee B. Global prevalence of post COVID-19 condition or long COVID: a meta-analysis and systematic review. J Infect Dis. (2022) 226(9):1593–1607. 10.1093/infdis/jiac136 35429399 PMC9047189

[9] Symptoms Overview | Altea Network [Internet]. [citado 3 de outubro de 2022]. Disponível em: Available at: https://www.altea-network.com/en/long-covid/symptoms-overview/

[10] Pinzon RT Wijaya VO Jody AA Nunsio PN Buana RB. Persistent neurological manifestations in long COVID-19 syndrome: a systematic review and meta-analysis. J Infect Public Health. (2022) 15(8):856–69. 10.1016/j.jiph.2022.06.013 35785594 PMC9221935

[11] Healey Q Sheikh A Daines L Vasileiou E. Symptoms and signs of long COVID: a rapid review and meta-analysis. J Glob Health. (2022) 12:05014. 10.7189/jogh.12.05014 35596571 PMC9125197

[12] Yelin D Moschopoulos CD Margalit I Gkrania-Klotsas E Landi F Stahl JP ESCMID rapid guidelines for assessment and management of long COVID. Clin Microbiol Infect. (2022) 28(7):955–72. 10.1016/j.cmi.2022.02.018 35182760 PMC8849856

[13] Michelen M Manoharan L Elkheir N Cheng V Dagens A Hastie C Characterising long COVID: a living systematic review. BMJ Glob Health. (2021) 6(9):e005427. 10.1136/bmjgh-2021-005427 34580069 PMC8478580

[14] Ceban F Ling S Lui LMW Lee Y Gill H Teopiz KM Fatigue and cognitive impairment in post-COVID-19 syndrome: a systematic review and meta-analysis. Brain Behav Immun. (2022) 101:93–135. 10.1016/j.bbi.2021.12.020 34973396 PMC8715665

[15] Sudre CH Murray B Varsavsky T Graham MS Penfold RS Bowyer RC Attributes and predictors of long COVID. Nat Med. (2021) 27(4):626–31. 10.1038/s41591-021-01292-y 33692530 PMC7611399

[16] Lopez-Leon S Wegman-Ostrosky T Perelman C Sepulveda R Rebolledo PA Cuapio A More than 50 long-term effects of COVID-19: a systematic review and meta-analysis. Sci Rep. (2021) 11(1):16144. 10.1038/s41598-021-95565-8 34373540 PMC8352980

[17] Anaya JM Rojas M Salinas ML Rodríguez Y Roa G Lozano M Post-COVID syndrome. A case series and comprehensive review. Autoimmun Rev. (2021) 20(11):102947. 10.1016/j.autrev.2021.102947 34509649 PMC8428988

[18] Fernández-de-Las-Peñas C Liave-Rincón AI Ortega-Santiago R Ambite-Quesada S Gómez-Mayordomo V Cuadrado ML Prevalence and risk factors of musculoskeletal pain symptoms as long-term post-COVID sequelae in hospitalized COVID-19 survivors: a multicenter study. Pain. (2022) 163(9):e989–96. 10.1097/j.pain.0000000000002564 34913880

[19] Khoja O Silva Passadouro B Mulvey M Delis I Astill S Tan AL Clinical characteristics and mechanisms of musculoskeletal pain in long COVID. J Pain Res. (2022) 15:1729–48. 10.2147/JPR.S365026 35747600 PMC9212788

[20] Magro C Mulvey JJ Berlin D Nuovo G Salvatore S Harp J Complement associated microvascular injury and thrombosis in the pathogenesis of severe COVID-19 infection: a report of five cases. Transl Res. (2020) 220:1–13. 10.1016/j.trsl.2020.04.007 32299776 PMC7158248

[21] Oudkerk M Büller HR Kuijpers D van Es N Oudkerk SF McLoud T Diagnosis, prevention, and treatment of thromboembolic complications in COVID-19: report of the national institute for public health of the Netherlands. Radiology. (2020) 297(1):E216–22. 10.1148/radiol.2020201629 32324101 PMC7233406

[22] Wang CC Chao JK Chang YH Chou CL Kao CL. Care for patients with musculoskeletal pain during the COVID-19 pandemic: physical therapy and rehabilitation suggestions for pain management. J Chin Med Assoc. (2020) 83(9):822–4. 10.1097/JCMA.0000000000000376 32618600 PMC7434017

[23] Fernández-de-Las-Peñas C Palacios-Ceña D Gómez-Mayordomo V Florencio LL Cuadrado ML Plaza-Manzano G Prevalence of post-COVID-19 symptoms in hospitalized and non-hospitalized COVID-19 survivors: a systematic review and meta-analysis. Eur J Intern Med. (2021) 92:55–70. 10.1016/j.ejim.2021.06.009 34167876 PMC8206636

[24] Disser NP De Micheli AJ Schonk MM Konnaris MA Piacentini AN Edon DL Musculoskeletal consequences of COVID-19. J Bone Joint Surg Am. (2020) 102(14):1197–204. 10.2106/JBJS.20.00847 32675661 PMC7508274

[25] Omar IM Weaver JS Samet JD Serhal AM Mar WA Taljanovic MS. Musculoskeletal manifestations of COVID-19: currently described clinical symptoms and multimodality imaging findings. Taljanovic RadioGraphics. (2022) 42(5):1415–32. 10.1148/rg.220036 35867593 PMC9341171

[26] Halpin SJ McIvor C Whyatt G Adams A Harvey O McLean L Postdischarge symptoms and rehabilitation needs in survivors of COVID-19 infection: a cross-sectional evaluation. J Med Virol. (2021) 93(2):1013–22. 10.1002/jmv.26368 32729939

[27] Van Kessel SAM Olde Hartman TC Lucassen PLBJ van Jaarsveld CHM. Post-acute and long-COVID-19 symptoms in patients with mild diseases: a systematic review. Fam Pract. (2022) 39(1):159–67. 10.1093/fampra/cmab076 34268556 PMC8414057

[28] Akbarialiabad H Taghrir MH Abdollahi A Ghahramani N Kumar M Paydar S Long COVID, a comprehensive systematic scoping review. Infection. (2021) 49(6):1163–86. 10.1007/s15010-021-01666-x 34319569 PMC8317481

[29] D’Ascanio L Pandolfini M Cingolani C Latini G Gradoni P Capalbo M Olfactory dysfunction in COVID-19 patients: prevalence and prognosis for recovering sense of smell. Otolaryngol Head Neck Surg. (2021) 164(1):82–6. 10.1177/0194599820943530 32662745

[30] Jacobs LG Gourna Paleoudis E Lesky-Di Bari D Nyirenda T Friedman T Gupta A Persistence of symptoms and quality of life at 35 days after hospitalization for COVID-19 infection. PLoS One. (2020) 15(12):e0243882. 10.1371/journal.pone.0243882 33306721 PMC7732078

[31] Tenforde MW Kim SS Lindsell CJ Billig Rose E Shapiro NI Files DC Symptom duration and risk factors for delayed return to usual health among outpatients with COVID-19 in a multistate health care systems network—United States. MMWR Morb Mortal Wkly Rep. (2020) 69(30):993–8. 10.15585/mmwr.mm6930e1 32730238 PMC7392393

[32] Xiong Q Xu M Li J Liu Y Zhang J Xu Y Clinical sequelae of COVID-19 survivors in Wuhan, China: a single-centre longitudinal study. Clin Microbiol Infect. (2021) 27(1):89–95. 10.1016/j.cmi.2020.09.023 32979574 PMC7510771

[33] Karcioglu O Topacoglu H Dikme O Dikme O. A systematic review of the pain scales in adults: which to use? Am J Emerg Med. (2018) 36(4):707–14. 10.1016/j.ajem.2018.01.008 29321111

[34] Fitch MI Bunston T Bakker D Mings D Sevean P. The fatigue pictogram: psychometric evaluation of a new clinical tool. Can Oncol Nurs J. (2011) 21(4):205–17. 10.5737/1181912x214205210 22216735

[35] Mota DdF Pimenta CdM Fitch MI. Pictograma de fadiga: uma alternativa para avaliação da intensidade e impacto da fadiga. Rev esc Enferm USP. (2009) 43(spe):1080–7. 10.1590/S0080-62342009000500012

[36] Berg KO Wood-Dauphinee SL Williams JI Maki B. Measuring balance in the elderly: validation of an instrument. Can J Public Health. (1992) 83(Suppl 2):S7–11. 1468055

[37] de Sousa KCA Gardel DG Lopes AJ. Postural balance and its association with functionality and quality of life in non-hospitalized patients with post-acute COVID-19 syndrome. Physiother Res Int. (2022) 27(4):e1967. 10.1002/pri.1967 35842844 PMC9349853

[38] St M ILJ Ko B Lr R JN. Brazilian version of the berg balance scale. Braz J Med Biological Res. (2004) 37(9):1411–21. 10.1590/s0100-879x2004000900017 15334208

[39] Klok FA Boon GJAM Barco S Endres M Geelhoed JJM Knauss S The post-COVID-19 functional status scale: a tool to measure functional status over time after COVID-19. Eur Respir J. (2020) 56(1):2001494. 10.1183/13993003.01494-2020 32398306 PMC7236834

[40] Machado FVC Meys R Delbressine JM Vaes AW Goërtz YMJ van Herck M Construct validity of the post-COVID-19 functional Status scale in adult subjects with COVID-19. Health Qual Life Outcomes. (2021) 19(1):40. 10.1186/s12955-021-01691-2 33536042 PMC7856622

[41] Watson MJ. Refining the ten-metre walking test for use with neurologically impaired people. Physiotherapy. (2002) 88(7):386–97. 10.1016/S0031-9406(05)61264-3

[42] Soares FHC Kubota GT Fernandes AM Hojo B Couras C Costa BV Prevalence and characteristics of new-onset pain in COVID-19 survivours, a controlled study. Eur J Pain. (2021) 25(6):1342–54. 10.1002/ejp.1755 33619793 PMC8013219

[43] Huang C Huang L Wang Y Li X Ren L Gu X 6-month consequences of COVID-19 in patients discharged from hospital: a cohort study. Lancet. (2021) 397(10270):220–32. 10.1016/S0140-6736(20)32656-8 33428867 PMC7833295

[44] Ojeda A Calvo A Cuñat T Artigas RM Comino-Trinidad O Aliaga J Rationale and study design of an early care, therapeutic education, and psychological intervention program for the management of post-intensive care syndrome and chronic pain after COVID-19 infection (PAIN-COVID): study protocol for a randomized controlled trial. Trials. (2021) 22:486. 10.1186/s13063-021-05463-7 34303381 PMC8310406

[45] Pérez-González A Araújo-Ameijeiras A Fernández-Villar A Crespo M Poveda E. Cohort COVID-19 of the galicia sur health research institute. Long COVID in hospitalized and non-hospitalized patients in a large cohort in northwest Spain, a prospective cohort study. Sci Rep. (2022) 12(1):3369. 10.1038/s41598-022-07414-x 35233035 PMC8888560

[46] Bakılan F Gökmen İG Ortanca B Uçan A Eker Güvenç Ş Şahin Mutlu F Musculoskeletal symptoms and related factors in postacute COVID-19 patients. Int J Clin Pract. (2021) 75(11):e14734. 10.1111/ijcp.14734 34387911 PMC8420386

[47] Long Q Li J Hu X Bai Y Zheng Y Gao Z. Follow-ups on persistent symptoms and pulmonary function among post-acute COVID-19 patients: a systematic review and meta-analysis. Front Med (Lausanne). (2021) 8:702635. 10.3389/fmed.2021.702635 34540862 PMC8448290

[48] Almas T Malik J Alsubai AK Jawad Zaidi SM Iqbal R Khan K Post-acute COVID-19 syndrome and its prolonged effects: an updated systematic review. Ann Med Surg (Lond). (2022) 80:103995. 10.1016/j.amsu.2022.103995 35721785 PMC9197790

[49] Kosilek RP Schmidt K Baumeister SE Gensichen J. Frequency and risk factors of post-intensive care syndrome components in a multicenter randomized controlled trial of German sepsis survivors. J Crit Care. (2021) 65:268–73. 10.1016/j.jcrc.2021.07.006 34280656

[50] Griffiths J Hatch RA Bishop J Morgan K Jenkinson C Cuthbertson BH An exploration of social and economic outcome and associated health-related quality of life after critical illness in general intensive care unit survivors: a 12-month follow-up study. Crit Care. (2013) 17(3):R100. 10.1186/cc12745 23714692 PMC3706775

[51] Giardini M Arcolin I Guglielmetti S Godi M Capelli A Corna S. Balance performance in patients with post-acute COVID-19 compared to patients with an acute exacerbation of chronic obstructive pulmonary disease and healthy subjects. Int J Rehabil Res. (2022) 45(1):47–52. 10.1097/MRR.0000000000000510 34860732 PMC8828308

[52] Pant P Joshi A Basnet B Shrestha BM Bista NR Bam N Prevalence of functional limitation in COVID-19 recovered patients using the post COVID-19 functional Status scale. JNMA J Nepal Med Assoc. (2021) 59(233):7–11. 10.31729/jnma.5980 34508442 PMC7893391

